# Host-adaptation of the rare *Enterocytozoon bieneusi* genotype CHN4 in *Myocastor coypus* (Rodentia: Echimyidae) in China

**DOI:** 10.1186/s13071-020-04436-0

**Published:** 2020-11-16

**Authors:** Fuchang Yu, Yangwenna Cao, Haiyan Wang, Qiang Liu, Aiyun Zhao, Meng Qi, Longxian Zhang

**Affiliations:** 1 College of Animal Science, Tarim University, No. 1188 Junken Avenue, Alar, 843300 Xinjiang People’s Republic of China; 2 College of Animal Science and Veterinary Medicine, Henan Agricultural University, No. 15 Longzihu University Area, Zhengzhou New District, Zhengzhou, 450046 Henan People’s Republic of China; 3 Experimental and Research Center, Henan University of Animal Husbandry and Economy, Zhengzhou, 450046 Henan People’s Republic of China

**Keywords:** Microsporidia, Rodent, Species specificity, Transmission, Zoonotic

## Abstract

**Background:**

*Enterocytozoon bieneusi* is a zoonotic gastrointestinal pathogen and can infect both humans and animals. The coypu (*Myocastor coypus*) is a semi-aquatic rodent, in which few *E. bieneusi* infections have been reported and the distribution of genotypes and zoonotic potential remains unknown.

**Methods:**

A total of 308 fresh fecal samples were collected from seven coypu farms in China to determine the infection rate and the distribution of genotypes of *E. bieneusi* from coypus using nested-PCR amplification of the internal transcribed spacer (ITS) region of the ribosomal RNA (rRNA) gene.

**Results:**

*Enterocytozoon bieneusi* was detected with an infection rate of 41.2% (*n* = 127). Four genotypes were identified, including three known genotypes (CHN4 (*n* = 111), EbpC (*n* = 8) and EbpA (*n* = 7)) and a novel genotype named CNCP1 (*n* = 1).

**Conclusions:**

The rare genotype CHN4 was the most common genotype in the present study, and the transmission dynamics of *E. bieneusi* in coypus were different from other rodents. To the best of our knowledge, this is the first report of *E. bieneusi* infections in coypus in China. Our study reveals that *E. bieneusi* in coypus may be a potential infection source to humans. 
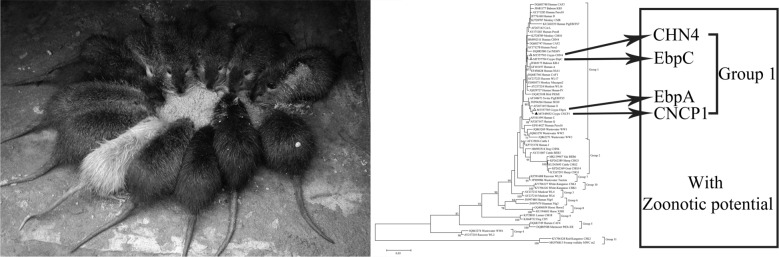

## Background

*Enterocytozoon bieneusi* is an obligate intracellular pathogen, which has been detected in a broad range of hosts, including humans, livestock, companion animals, birds and wildlife [1, 2]. Hosts can be infected by ingesting infective spores through food-borne and water-borne routes or direct contact with infected humans or animals [3]. To date, over 500 genotypes of *E. bieneusi* were identified in the world by molecular genotyping based on internal transcribed spacer (ITS) region of the ribosomal RNA (rRNA) gene [1, 4]. These genotypes were divided into 11 distinct groups (groups 1 to 11) in a phylogenetic analysis [5]. The majority of the zoonotic genotypes are clustered in Group 1 [5]. Meanwhile, more and more reports show that some genotypes (I, J, BEB4 and BEB6) in Group 2 can also infect humans, indicating a low host specificity and zoonotic inherence of this group [1, 6, 7]. Other groups mostly contain host-adapted genotypes [6].

Previous studies indicated that at least 63 *E. bieneusi* genotypes have been identified in more than 20 rodent species, including zoonotic ones (BEB6, C, D, EbpA, EbpC, H, Peru8, Peru11, Peru16, PigITS5, S6 and Type IV) [1, 8, 9]. In a previous study, the zoonotic transmission of *E. bieneusi* occurred between a child and guinea pigs in Peru [10]. About 40% to 50% of the mammalian species are rodents, which are distributed throughout the world except the Antarctic and a handful of islands [11]. Because of their abundant population and broad active range, rodents infected with *E. bieneusi* pose an unneglectable threat to public health. The coypu (*Myocastor coypus*) is a large rodent adapted to amphibious environments; nowadays coypus are being widely raised in farms as important fur-bearing animals. However, there is limited information about the infection rate and genetic characteristics of *E. bieneusi* in coypus worldwide. Therefore, this study aimed to determine the genotypes and infection rate and assess the zoonotic potential of *E. bieneusi* from coypus in China.

## Methods

### Sample collection

**Fig. 1:**
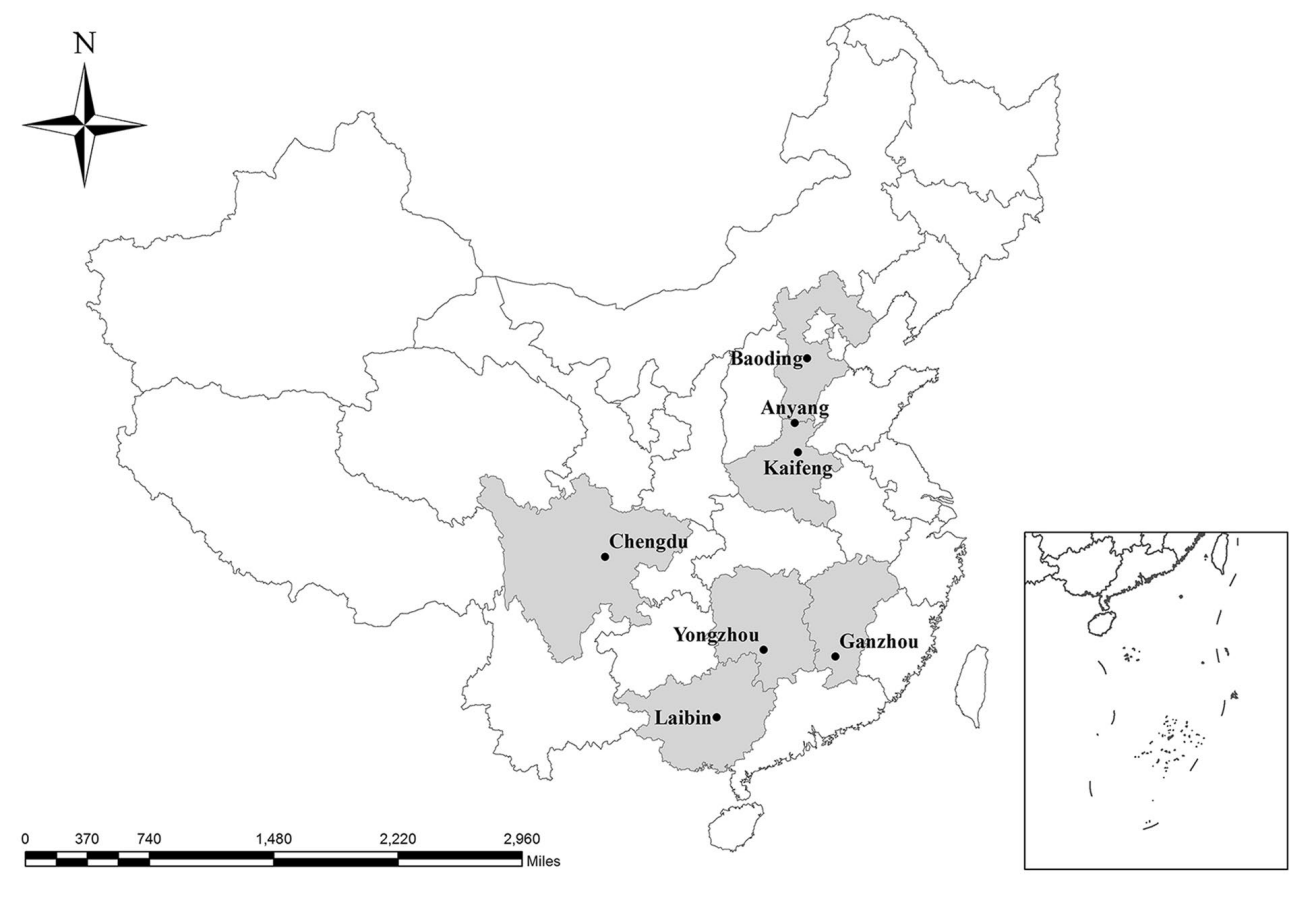
Map of the Peopleʼs Republic of China showing the sampling locations. The figure was originally designed by the authors under the software ArcGIS 10.2. The original vector diagram imported in ArcGIS was adapted from Natural Earth (https://www.naturalearthdata.com)

A total of 308 fresh fecal samples were collected from asymptomatic coypus from seven farms in Anyang and Kaifeng in Henan Province, Yongzhou in Hunan Province, Laibin in Guangxi Zhuang Autonomous Region, Baoding in Hebei Province, Chengdu in Sichuan Province and Ganzhou in Jiangxi Province in China (Table 1, Fig. 1). Each farm was sampled on one occasion from August 2018 to March 2019. In each farm, about 2–4 coypus were kept in one accommodation, which was surrounded by 80 cm-high walls to fence the animals off from each other. The ground of the accommodation was hardened with cement. An accommodation is typically composed of a piece of vacant land as the playground and a pool in which the coypus can swim. The samples were collected when the handlers finished the ground using a high-pressure water gun. All the fecal samples were collected immediately after they excreted using sterile polyethylene gloves and marked with animal information. To avoid duplicate sampling of animals, only one fecal sample was collected from one location of the ground in each accommodation, and all deposits from each accommodation pooled as a single sample. All the samples were transferred to the laboratory in a cooler with ice packs within 36 h and stored at 4 °C.

**Table 1:** Distribution of *E. bieneusi* genotypes in coypus from different farms in China

Location	No. of sample	No. of positive	Infection rate (95% CI) (%)	Genotype (*n*)
Anyang	101	73	72.3 (63.0–81.5)	CHN4 (73)
Baoding	35	22	62.9 (45.3–80.3)	CHN4 (22)
Chengdu	40	6	15.0 (2.7–27.3)	CHN4 (6)
Ganzhou	35	7	20.0 (5.3–34.7)	CHN4 (7)
Kaifeng	52	16	30.8 (17.3–44.3)	CHN4 (2), EbpA (7), EbpC (6), CNCP1 (1)
Laibin	22	2	9.1 (0–23.4)	CHN4 (1), EbpC (1)
Yongzhou	23	1	4.3 (0–14.8)	EbpC (1)
Total	308	127	41.2 (35.6–46.9)	CHN4 (111), EbpA (7), EbpC (8), CNCP1 (1)

### DNA extraction and PCR amplification

Genomic DNA (gDNA) was directly extracted from 200 mg of each sample using E.Z.N.A. Stool DNA Kit (Omega Biotek Inc., Norcross, GA, USA) according to the manufacturer’s protocol with minor modification.

All samples were tested using a nested PCR that targets ITS region (~389-bp fragment) of the rRNA gene of *E. bieneusi* using primers described previously by Sulaiman et al. [12]. Double distilled water and known positive DNA derived from a golden snub-nosed monkey (genotype D, GenBank: KU604932) were used as negative and positive controls, respectively. The secondary PCR products were separated electrophoretically on 1% agarose (Life Technologies Corporation, CA, USA) gel stained with DNAGreen (Tiandz, Beijing, China) and visualized under UV light.

### Sequencing and data analyses

Positive secondary PCR products were sequenced bidirectionally by Sangon Biotech Co. Ltd., Shanghai, China. The sequences obtained here were assembled and edited in the software Lasergene EditSeq version 7.1.0 (https://www.dnastar.com/) and multiple alignment with the reference sequences downloaded from GenBank was applied in Clustal X version 2.1 (https://www.clustal.org/).

All statistical analyses were performed with IBM SPSS Statistics version 19.0 (www.ibm.com/products/spssstatistics). Difference of prevalence of *E. bieneusi* among different age groups were compared using Fisher’s exact test, and the odds ratios (ORs) with the 95% confidence interval (CI) were also calculated. A two-sided *P*-value of 0.05 or less was set as significant.

To reveal the phylogenetic relationships and zoonotic risk of *E. bieneusi* isolates, a phylogenetic tree was constructed by the Neighbor-Joining (NJ) method using the Kimura-2-parameter algorithm in MEGA version 7.0.26 (https://www.megasoftware.net). The robustness of the nodes was tested by a bootstrap analysis of 1000 iterations.

## Results

### Infection rate of E. bieneusi in coypus

*Enterocytozoon bieneusi* was detected in 127 of 308 coypus with an infection rate of 41.2%. This parasite was found in every farm, and the highest infection rate of *E. bieneusi* in coypus was detected in Anyang (72.3%, 73/101), followed by Baoding (62.9%, 22/35), Kaifeng (30.8%, 16/52), Ganzhou (20.0%, 7/35), Chengdu (15.0%, 6/40), Laibin (9.1%, 2/22) and Yongzhou (4.3%, 1/23) (Table 1). The differences in infection rates of *E. bieneusi* in coypus among different farms were statistically significant (*P* < 0.0001).

The highest infection rate (76.9%, 50/65) was detected in the < 3-month-old group, followed by the 3–6 month-old group (51.1%, 24/47) and > 6 month-old group (28.5%, 53/186) (Table 2) (*P* < 0.0001). The correlations between age and the infection rates were evaluated by calculating the ORs and their 95% CIs, which are shown in Table 2. There was a significant negative correlation between the infection rate and age in this study, as an OR of 0.31 (95% CI: 0.14–0.70, *P* = 0.005) was associated with the 3–6-month-old group, and an OR of 0.12 (95% CI: 0.06–0.23, *P* < 0.0001) was associated with the > 6-month-old group.

**Table 2:** Occurrence of *E. bieneusi* in coypus by age

Age (month)	No. of samples	Infection rate (95% CI) (%)	*P*-value	OR (95% CI)
< 3	65	76.9 (65.9–87.9)	< 0.0001	1.00
3–6	47	51.1 (35.7–66.4)	0.005	0.31 (0.14–0.70)
> 6	196	27.0 (20.1–33.5)	< 0.0001	0.11 (0.06–0.21)

### Enterocytozoon bieneusi ITS genotypes

Four distinct *E. bieneusi* genotypes, including three previously reported genotypes [CHN4 (*n* = 111), EbpC (*n* = 8), EbpA (*n* = 7)], and one novel genotype (named CNCP1, *n* = 1) were observed. Genotype CHN4 was the most common genotype and detected in 6 farms except the farm in Yongzhou. Genotype EbpC was distributed in Yongzhou, Laibin and Kaifeng, while genotype EbpA and novel genotype CNCP1 were only detected in the specimens from Kaifeng.

CHN4 was the only genotype detected in the < 3-month-old group (*n* = 50). In the 3–6-month-old group, CHN4 was also the predominant genotype, which was detected in 16 samples, followed by EbpA (*n* = 4), EbpC (*n* = 3) and CNCP1 (*n* = 1). In the age group > 6 months, 3 genotypes (CHN4, EbpC and EbpA) were detected in 45, 5 and 3 samples, respectively.

### Phylogenetic analysis of E. bieneusi

**Fig. 2:**
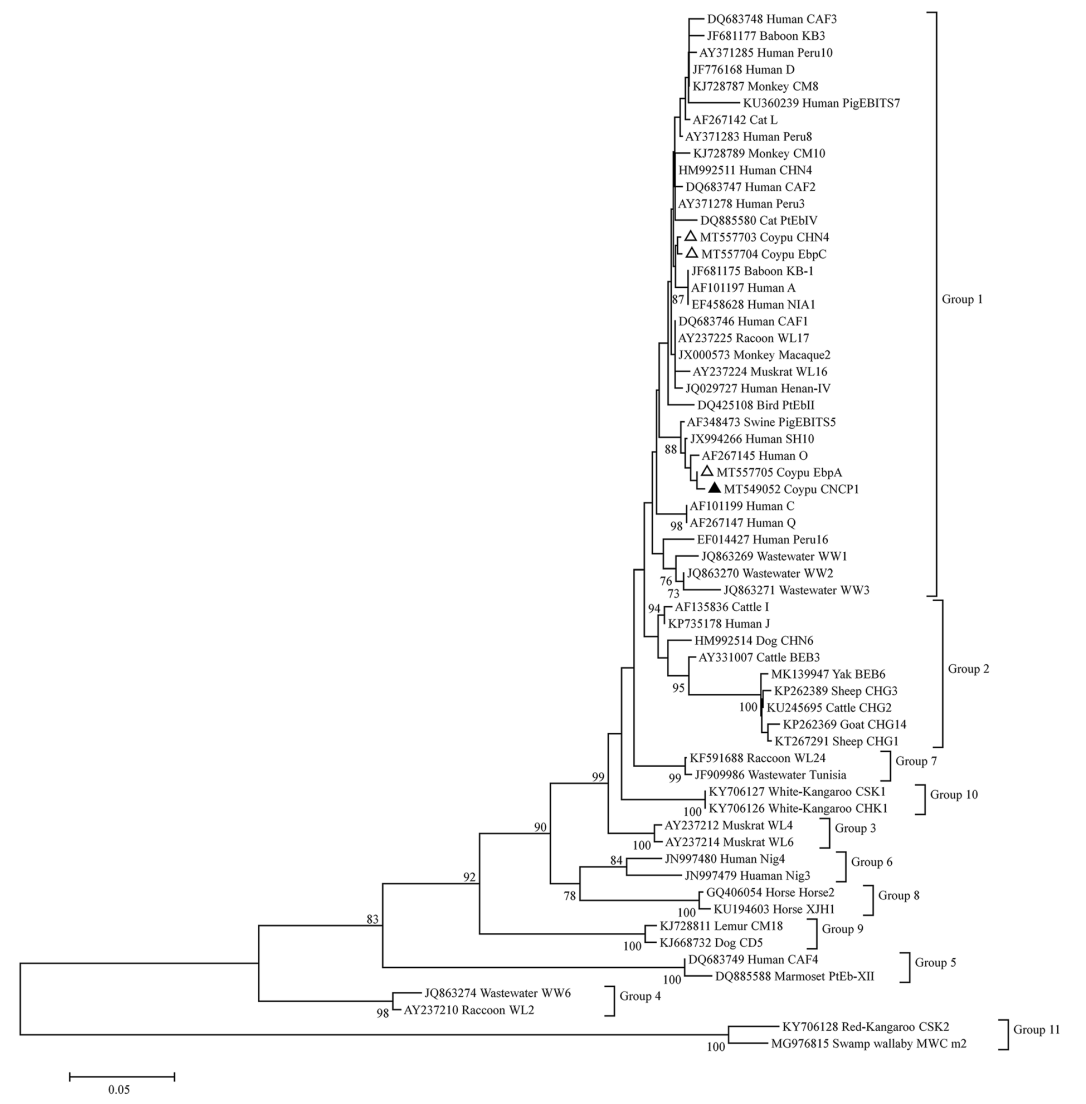
Neighbor-joining tree of *Enterocytozoon bieneusi* ITS genotypes. Phylogenetic relationships of *Enterocytozoon bieneusi* genotypes of this study and other genotypes previously deposited in GenBank. Bootstrap values > 50% from 1000 are shown on nodes. Sample names include GenBank accession number followed by host and then genotype designation. Known and novel genotypes identified in this study are indicated by empty and filled triangles, respectively

The phylogenetic relationships and zoonotic risk of *E. bieneusi* genotypes were analyzed by the NJ tree. Genotype CNCP1 had one single nucleotide polymorphism (SNP) at nucleotide position 274 (G to A) compared to genotype EbpA (GenBank: MK968834). All the genotypes identified in this study were clustered in Group 1 (Fig. 2).

## Discussion

The infection rate of *E. bieneusi* in rodent species varies from 2.5% to 100% worldwide [13, 14]. To the best of our knowledge, this is the first report of *E. bieneusi* infections in coypus in China. In the present study, the overall infection rate of *E. bieneusi* was 41.2% in coypus, which is higher than the infection rate of *E. bieneusi* reported in brown rats (7.9%) [8], bamboo rats (5.1%) [15], experimental brown rats (4.8%) [16], commensal rodents (mouse and brown rat) (4.0%) [14], pet chinchillas (3.6%) [17] and red squirrels (19.4%) [18] in China. In addition, lower infection rates were also reported in wild house mice (10.7%) from a hybrid zone across the Czech Republic-Germany border [19], and beavers (15.3%) and muskrats (8.4%) from the USA [20]. However, higher infection rates of *E. bieneusi* were reported in chipmunks (71.4%) and woodchucks (100%) from USA [13]. Similar infection rates of *E. bieneusi* have been reported in small rodents (mouse, bank vole, yellow-necked mouse and striped field mouse) (38.9%) from southwestern Poland [21], and a laboratory prairie dog colony (37.9%) in the USA [22]. The infection rates of *E. bieneusi* in rodents could be influenced by many factors, such as animal immune status, age distribution, sample size, detection method, feeding environment, management system and population density [16]. Because the high infection rate detected in coypus in our study, we can draw a preliminary inference that coypus are more susceptible to *E. bieneusi* than many other rodent species, which should be confirmed by more investigations in the future.

A variation of the positive rate of *E. bieneusi* in coypus was observed in the present study with the highest being detected in Anyang (72.3%, 73/101) and the lowest in Laibin (9.1%, 2/22). Geographical location-based variation in the prevalence of *E. bieneusi* in rodents has been reported such as in brown rats in different provinces in China, which was ranged between 2.9–14.7% [8, 14, 16, 23, 24]. This phenomenon has also been reported in other animals, for example, in alpacas (*Vicugna pacos*) in China (0–42.9%) [25] and in Asiatic black bear (*Ursus thibetanus*) in China (0–50%) [26]. The difference may be related to geographical environments and feeding density.

In the present study, the dominant genotype of *E. bieneusi* was CHN4, which was detected in six cities except Yongzhou, indicating that genotype CHN4 is commonly found in coypus in China. This genotype has been identified in three human and two cattle samples [27] and four pre-weaned calf samples [28] from China, and is found for the first time in coypus in the present study. These findings indicated that genotype CHN4 has a wide range of animal reservoirs and potential for zoonotic transmission. Genotype D was identified in squirrels from China [29] and USA [13], chipmunks [30], bamboo rats [15] and brown rats [8, 23] from China, house mice from Czech Republic-Germany border [19] and striped field mice from Poland [21], and genotype WL4 was observed in squirrels, chipmunks and muskrats from the USA [13, 20] (Table 3). EbpA, EbpC, PigEBITS7, S7, Peru16 and CHG14 have also been reported as the most common genotypes in experimental brown rat, beaver, giant rat, guinea pig, guinea pig and brown rat, respectively [10, 14, 16, 20, 23, 31]. Additionally, in a more recent study of *E. bieneusi* in Himalayan marmots (*Marmota himalayana*) and Alashan ground squirrels (*Spermophilus alashanicus*) revealed that genotype ZY37 was the most common one [9]. The rare genotype CHN4 was the dominant genotype, indicating that the transmission dynamic of *E. bieneusi* in coypus is different from other rodents. This may be explained by the unique life habits of coypus as aquatic rodents compared to other rodents involved in previous studies.

**Table 3:** Prevalence and genotype distribution of *Enterocytozoon bieneusi* in rodents worldwide (Li et al. [1])

Host	Location	Infection rate (%) (No. positive/Total no.)	Genotype (*n*)	References
Alashan ground squirrel	China	3.0 (3/99)	HN39 (1), HN96 (1), YAK1 (1)	Xu et al. [9]
Chipmunk	USA	71.4 (5/7)	WL4 (3), Type IV (1), WL23 (1)	Guo et al. [13]
	China	17.6 (49/279)	D (6), Nig7 (4), CHG9 (2), CHY1 (5), SCC-1 (17), SCC-2 (9), SCC-3 (5), SCC-4 (1)	Deng et al. [30]
Eastern gray squirrel	USA	29.7 (11/37)	WL4 (5), Type IV (3), PtEb V (1), WL21 (1), WW6 (2)	Guo et al. [13]
Himalayan marmot	China	11.8 (47/399)	ZY37 (27), YAK1 (17), SN45 (1), XH47 (1), ZY83 (1)	Xu et al. [9]
Prairie dog	USA	48.3 (14/29)	Row^a^ (14)	Roellig et al. [22]
Red-bellied tree squirrel	China	16.7 (24/144)	D (18), EbpC (3), SC02 (1), CE01 (1), horse2 (1)	Deng et al. [29]
	China	4.2 (1/24)	D (1)	Zhao et al. [24]
Red squirrels	China	19.4 (61/314)	D (27), SCC-2 (18), SCC-4 (12), RS01 (2), RS02 (2)	Deng et al. [18]
Woodchuck	USA	100 (5/5)	Type IV (1)^b^, WL20 (1), WL4 (2), WL22 (1, WW6 (1)	Guo et al. [13]
Asian house rat	China	23.1 (31/134)	PigEbITS7 (16), D (12), ESH-02 (1), Type-IV (1), EbpA (1)	Zhao et al. [24]
Brown rat	China	7.9 (19/242)	D (17), Peru6 (2)	Zhao et al. [8]
	China	2.5 (7/277)	CHG14(3), BEB6(2), D(1), CHG2(1)	Yu et al. [14]
	China	17.2 (17/152)	D (12), Peru11(3), S7 (1), SCC-2 (1)	Wang et al. [23]
	China	14.3 (8/56)	D (3), PigEbITS7 (1), Type IV (1), Peru 8 (1), HNR-I (1), HNR-II (1)	Zhao et al. [24]
	China	4.8 (14/291)	EbpA (7), EbpC (3), CHY1 (2), N (1), SHR1 (1)	Li et al. [16]
Chinese white-bellied rat	China	18.2 (6/33)	D (3), PigEBITS7 (2), Type-IV (1)	Zhao et al. [24]
Deer mouse	USA	23.6 (13/55)	WL4 (10), WL23 (2), WL25 (1)	Guo et al. [13]
Edwardʼs long-tailed rat	China	7.9 (3/38)	D (2), HNR-III (1)	Zhao et al. [24]
House mouse	China	3.2 (1/31)	D (1)	Yu et al. [14]
	Czech/German border	10.7 (31/289)	D (10), PigEBITS5 (7), CZ3 (4), Peru8 (4), C (2), EbpA (2), H (1), S6 (1)	Sak et al. [19]
	Poland	28.6 (6/21)	WR3 (1)	Perec-Matysiak et al. [21]
Indo-Chinese forest rat	China	9.3 (5/54)	D (3), Type-IV (1), HNR-III (1)	Zhao et al. [24]
Lesser rice-field rat	China	36.4 (16/44)	HNR-VII (15), D (1)	Zhao et al. [24]
Striped field mouse	Poland	42.9 (79/184)	D (6), gorilla 1 (1), WR5 (1), WR8 (2), WR7 (1)	Perec-Matysiak et al. [21]
Yellow-necked mouse	Poland	30.0 (18/60)	D (2), WR1 (1), WR4 (1), WR6 (6), WR9 (1)	Perec-Matysiak et al. [21]
White-toothed rat/giant rat	China	33.3 (76/228)	PigEBITS7 (22), D (14), K (8), Peru8 (2), CQR1 (10), CQR2 (15), CQR3 (1), GDR1(2), GDR2 (1)	Gui et al. [31]
Bank vole	Poland	39.1 (18/46)	D (2), WR2 (1), WR6 (2), WR10 (2)	Perec-Matysiak et al. [21]
Muskrat	USA	8.4 (20/239)	WL4 (8), WL15 (4), EbpC (3), D (2), WL10 (1), WL14 (1), WL6 (1)	Sulaiman et al. [20]
Vole	USA	26.7 (4/15)	Peru11 (2), WL21(2), type IV (1), WL20 (1)	Guo et al. [13]
Bamboo rat	China	5.1 (22/435)	D (17), J (1), BR1 (1), BR2 (1), EbpA (1), PigEBITS7 (1)	Wang et al. [15]
	China	15.4 (18/117)	D (15), Peru 11 (1), HNR-IV (1), HNR-V(1)	Zhao et al. [24]
Beaver	USA	15.3 (13/85)	EbpC (5), D (4), WL7, WL9, WL12, and WL15 (1 each)	Sulaiman et al. [20]
Chinchilla	China	3.6 (5/140)	D (2), BEB6 (3)	Qi et al. [17]
Asiatic brush-tailed porcupine	China	7.5 (7/93)	D (3), HNR-VI (2), S7 (1), CHG5 (1)	Zhao et al. [24]
Guinea pig	Peru	14.9 (10/67)	Peru16 (10)	Cama et al. [10]
	China	20.2 (35/173)	S7 (30), PGP (5)	Wang et al. [23]

^a^Invalid genotype

^b^One sample was co-infected with Type IV and WL20

Genotype EbpA and EbpC have been detected in several rodent species (squirrel, house mouse, experimental brown rat, muskrat, bamboo rat and beaver) worldwide [15, 16, 19, 20, 29] (Table 3). They are two of the most common genotypes detected in both immunocompetent and immunocompromised people worldwide [1]. Meanwhile, genotype EbpA and EbpC have a vast host range, such as non-human primates (NHPs), livestock (cattle, buffalo, sheep and goat), pets (dog and horse), wild animals (deer, fox, raccoon, bear, panda and otter) and birds (pigeon, crane and parrot) [1]. These two genotypes also have been observed in lake water [32], river water [33] and wastewater treatment plants [34, 35]. According to these data, the interspecies transmission of genotype EbpA and EbpC pose a zoonotic risk to human or other animals, and coypus may serve as a reservoir of EbpA and EbpC in the *E. bieneusi* transmission.

In the phylogenetic analysis, an NJ tree was constructed and the novel genotype CNCP1 clustered with CHN4, EbpC and EbpA in group 1. The majority of the zoonotic genotypes belongs to the Group 1, and genotypes CHN4, EbpC and EbpA have been reported in humans [27, 36, 37], indicating that genotype CNCP1 maybe has zoonotic potential and the *E. bieneusi* isolates in coypus detected in this study can be transmissible from coypus to humans, especially the animal handlers, or *vice versa*.

## Conclusions

*Enterocytozoon bieneusi* infection was highly observed in coypus from China, with the high prevalence of rare genotype CHN4. The presence of zoonotic genotypes EbpC and EbpA revealed the role of coypus as a reservoir of *E. bieneusi* and posed a threat to the public health. To further characterize the role of coypus in the transmission of microsporidiosis, more intensive research of *E. bieneusi* should be devised and employed.

## Data Availability

The nucleotide sequences from this study were deposited in the GenBank database under the accession numbers MT549052 and MT557703-MT557705.

## Abbreviations

ITS: Internal transcribed spacer
gDNA: Genomic DNA
CI: Confidence interval
OR: Odds ratio
NJ: Neighbor-joining
NHP: Non-human primate
SNP: Single-nucleotide polymorphism
rRNA: Ribosomal RNA
